# Factors driving higher opioid use after total hip arthroplasty: Insights from a large-scale, tertiary centre analysis

**DOI:** 10.1051/sicotj/2025064

**Published:** 2026-02-03

**Authors:** Andreas Fontalis, Shannon Tse, Mads K. Hansen, Adam T. Yasen, Crystallynn S. The, Isabella Catrina Haddad, Warran Wignadasan, Ricci Plastow, Fares S. Haddad

**Affiliations:** 1 Department of Trauma and Orthopaedic Surgery, University College Hospital London UK; 2 Division of Surgery and Interventional Science, University College London Gower Street London WC1E 6BT UK; 3 Department of Orthopaedic Surgery, University of California Davis Sacramento USA; 4 University College London Medical School London UK

**Keywords:** Opioid use, Total hip arthroplasty, Postoperative pain, Pain management, Risk stratification

## Abstract

*Introduction*: Effective postoperative pain management is imperative in total hip arthroplasty (THA) to enable early mobilization and accelerate recovery pathways. This study investigated the patterns of inpatient opioid consumption following THA and identified the factors associated with increased opioid usage. *Methods*: In this large-scale, single-institution study, we analyzed data from 1,867 primary THAs between April 2019 and July 2023. We collected data on demographics, length of stay (LOS), type of anaesthesia, Post Anaesthesia Care Unit (PACU) admissions, 30-day readmissions, total opioid consumption (MME; morphine milligram equivalents), implant fixation techniques, surgical characteristics and pre- and postoperative haemoglobin (Hb) levels. Factors associated with increased opioid consumption (patients in the ≥ 75th percentile of inpatient opioid consumption; MME ≥ 211.9 mg) were identified through univariate and multivariate logistic regression models. *Results*: The cohort included 1150 women (61.6%) and 717 men (38.4%). The median inpatient opioid use was 88 mg (IQR = 39.3–211.9). In the univariate model, significant predictors included age, American Society of Anaesthesiologists (ASA) score, manual THA technique, general anaesthesia, pre- and postoperative Hb levels, need for PACU admission and year of surgery. After adjusting for baseline demographics in the hierarchical multivariate logistic regression model, significant predictors of higher opioid utilization were age (OR 0.989 [95% CI 0.981–0.997], *p* = 0.01), general anaesthesia (OR 2.386 [95% CI 1.865–3.054], *p* < 0.001), PACU admission (OR 2.098 [95% CI 1.310–3.358], *p* = 0.002), ASA score (OR 1.492 [95% CI 1.193–1.866], *p* < 0.001), postoperative Hb levels (OR 0.981 [95% CI 0.970–0.992], *p* < 0.001), and year of surgery (OR 0.638 [95% CI 0.579–0.703], *p* < 0.001) indicating that later years were associated with lower odds of high opioid consumption). *Discussion*: Younger age, higher ASA scores, lower postoperative haemoglobin, the need for PACU admission and general anaesthesia were significantly associated with increased opioid consumption following THA. Recognizing these factors can facilitate the development of tailored postoperative pain management protocols, enabling targeted interventions that minimize opioid reliance while enhancing recovery.

## Introduction

Postoperative pain management remains a critical aspect of recovery following total hip arthroplasty (THA), despite advances in surgical techniques, implant technology, and overall patient outcomes. Effective pain control is essential in the postoperative period, as inadequate pain management can delay rehabilitation, prolong hospital stays, increase healthcare costs, and negatively impact patient satisfaction [1].

Postoperative opioid consumption is influenced by various factors, including patient-related factors such as demographics and comorbidities [2, 3], surgical characteristics [4], as well as the opioid doses prescribed by physicians [5–7]. Patients experiencing higher pain levels typically require larger doses of opioids following surgery [8, 9]. However, the extensive use of opioids in managing post-surgical pain, particularly in orthopaedic surgery, has contributed to the ongoing opioid epidemic. Increasing awareness of the risks associated with opioid use, such as tolerance, dependence, and addiction, has led to growing concerns about overprescription [10, 11]. Studies have shown that chronic opioid use following total joint arthroplasty is associated with increased postoperative complications and higher costs of care [11, 12].

Therefore, balancing effective pain relief while minimizing opioid-related adverse effects is essential. Identifying factors that influence opioid consumption is therefore crucial for optimizing postoperative care in THA patients and guiding targeted pain management protocols that reduce opioid usage without compromising patient outcomes. Multimodal analgesia, which combines different types of analgesics and techniques like non-steroidal anti-inflammatory medications (NSAIDs) and regional blocks, has emerged as an effective strategy in total joint arthroplasty to manage postoperative pain while minimizing opioid consumption [13, 14]. In addition to reducing morphine milligram equivalent (MME) requirements, multimodal approaches help lower the risk of opioid-related adverse drug effects such as nausea, vomiting, and altered mental status, which can improve patient comfort and enhance recovery following surgery.

There are several database studies on chronic postoperative opioid use following total joint arthroplasty [11, 12, 15, 16].

## Material and methods

**Figure 1:**
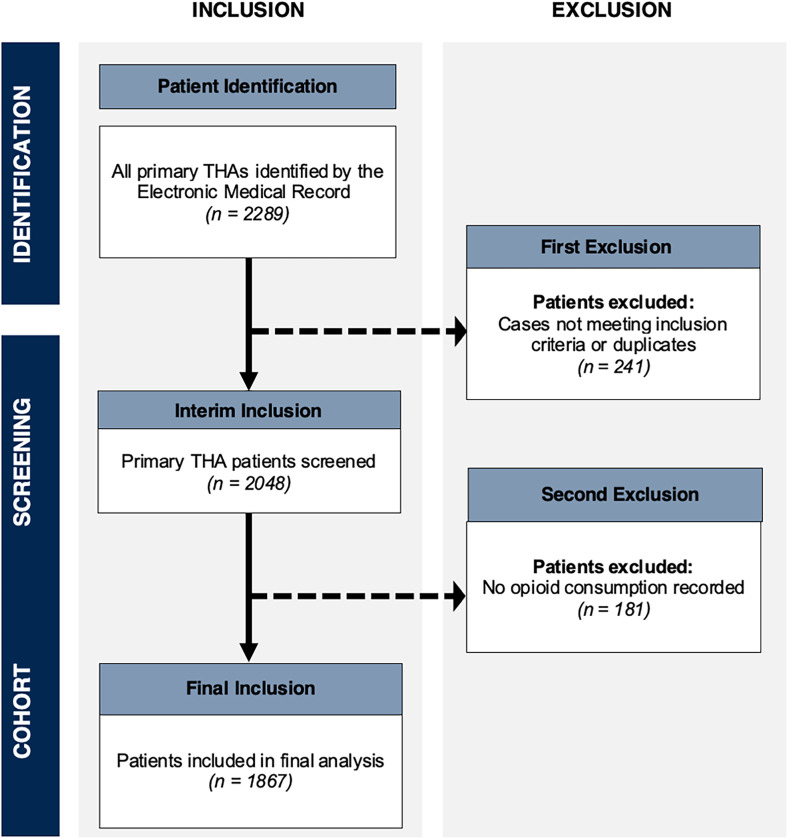
Flowchart showing the identification of cases and exclusions from the final analysis.

Patients of all ages undergoing primary THA for various indications, including osteoarthritis, osteonecrosis, inflammatory arthritis, and post-traumatic arthritis, were included in the study. All procedures were identified retrospectively from a prospectively maintained institutional database, using International Classification of Diseases (ICD-10) and OPCS (Office of Population Censuses and Surveys) procedure codes. The study period covered patients treated between April 2019 and July 2023 at a single tertiary academic centre (Figure 1). Revision surgeries and patients without a recorded morphine dose were excluded from the analysis, as opioid consumption could not be assessed. Postoperative inpatient opioid consumption (OC) was recorded and converted into morphine milligram equivalents (MME) based on the Faculty of Pain Medicine / Royal College of Anaesthetists’ Opioids Aware equi-analgesic guidance [17] and current BNF figures [18]. Conversions included oral and parenteral morphine, oxycodone, codeine, tramadol and fentanyl. Patients were divided into two groups based on their OC. High OC was defined as patients with inpatient MME ≥ 211.9 mg, representing the ≥ 75th percentile of the cohort; while the low/average OC group included all other patients. This percentile-based threshold was selected to account for the right-skewed distribution of opioid consumption, allowing identification of the highest-use quartile while maintaining adequate group sizes for multivariate modeling.

Patient characteristics including age, sex, body mass index (BMI), preoperative and postoperative haemoglobin (Hb) levels, the American Society of Anaesthesiologists (ASA) score, and the Index of Multiple Deprivation (IMD), which measures relative deprivation within the United Kingdom, were collected through review of medical records. Surgical characteristics included duration of surgery, anaesthesia type, implant fixation technique, and robotic or manual technique. Postoperative factors, including length of stay, requirement for Post Anaesthesia Care Unit (PACU) admission, and 30-day hospital readmissions, were recorded.

All robotic-assisted THAs were performed with the CT-guided semi-active Mako Robotic Arm Interactive Orthopaedic system (Stryker Corp., Mako Surgical Corp., Ft. Lauderdale, FL, USA). All patients underwent standardized rehabilitation with full weight-bearing and active range of movement exercises as per standard post-THA protocol. Paracetamol and NSAIDs (unless contraindicated) were prescribed routinely for all patients as part of our institutional multimodal analgesic protocol. Gabapentin and other adjunct non-opioid agents were not routinely used. Opioids were administered as rescue (PRN) analgesia for uncontrolled pain.

Institutional approval was obtained, and the study was registered as a quality improvement project with our surgical specialties division (registration number SS054).

Descriptive statistics were performed to analyze patient and surgical characteristics with the appropriate tests of significance applied. Normality was assessed using the Kolmogorov-Smirnov and Shapiro-Wilk tests, as well as skewness, kurtosis, and boxplots. Initially, binary univariate logistic regression was performed to identify individual predictors associated with high OC. Significant variables from the univariate analysis, along with clinically relevant parameters, were then included in a stepwise hierarchical multivariate logistic regression model to further assess predictors for high OC, adjusting for baseline demographics and potential confounders. Surgical duration was analysed per 10-minute increment. Analyses were conducted on a complete-case basis; no imputation was performed for missing data. Collinearity was assessed using variance inflation factors (VIFs) and all values were below 2.2, indicating acceptable levels of independence among variables. Statistical analyses were performed using SPSS Version 29 (IBM, Chicago, IL, USA), and a *p*-value of < 0.05 was considered statistically significant.

A total of 2048 primary THAs were initially identified for the study. After excluding patients without recorded opioid consumption data, 1,867 cases were included in the final analysis. The final cohort included 1150 women (61.6%) and 717 men (38.4%), with a median age of 64 years (IQR 54–73). Median BMI was 27.6 kg/m^2^ (IQR 24.3–32.0).

## Results

The overall median in-hospital opioid consumption MME was 88 mg (IQR 39.3–211.9). Patient characteristics are shown in Table 1. There was no difference in terms of age, gender, BMI, and IMD between the high and low/average OC groups. Patients in the high OC group had a higher proportion of ASA 3 and 4 scores (*P* < 0.001).

**Table 1:** Summary of patient characteristics.

	Overall	Low/average OC	High OC	*p*
Age (years), median (IQR)	64 (54–73)	65 (54–73)	62 (51–73)	*P* = 0.061
Female gender, *n* (%)	1150 (61.6%)	845 (60.4%)	305 (65%)	*P* = 0.077
BMI (kg/m^2^), median (IQR)	27.6 (24.3–32.0)	27.5 (24.3–31.6)	27.9 (24.2–33.1)	*P* = 0.232
Preoperative Hb (g/L), median (IQR)	134 (126–144)	134 (126–145)	133 (122–142)	***P* < 0.001**
Postoperative Hb (g/L), median (IQR)	114 (103–124)	114 (104–125)	111 (99–121)	***P* < 0.001**
Postoperative Hb drop (g/L), median (IQR)	20 (12–28)	20 (12–28)	21 (12–29)	*P* = 0.527
ASA score, *n* (%):				
1	207 (11.3%)	161 (11.7%)	46 (9.9%)	***P* < 0.001**
2	1204 (65.5%)	939 (68.3%)	265 (57.2%)	
3	408 (22.2%)	266 (19.4%)	142 (30.7%)	
4	18 (1.0%)	8 (0.6%)	10 (2.2%)	
IMD quintile, *n* (%)				
Q1	282 (15.6%)	211 (15.6%)	71 (15.5%)	*P* = 0.974
Q2	524 (28.9%)	389 (28.8%)	135 (29.5%)	
Q3	430 (23.7%)	326 (24.1%)	104 (22.7%)	
Q4	340 (18.8%)	254 (18.8%)	86 (18.8%)	
Q5	235 (13.0%)	173 (12.8%)	62 (13.5%)	

IQR, inter-quartile range; BMI, body mass index;- Hb, haemoglobin; ASA, American Society of Anaesthesiologists Score; IMD, Index of Multiple Deprivation. Bold indicates statistical significance (*p* < 0.05).

Surgical characteristics and postoperative factors are presented in Table 2. A higher proportion of high OC patients had surgery under general anaesthesia (*P* < 0.001) and received manual THA (*P* < 0.001). The low/average OC group had higher preoperative and postoperative haemoglobin compared to the high OC group (*P* < 0.001). There was no statistically significant difference between the groups in relation to postoperative haemoglobin drop (Table 1). A higher proportion of patients in the high OC group were admitted to PACU postoperatively (13.6% vs 5.4%, *P* < 0.001), and had a longer length of hospital stay (85 vs 56 hours, *P* < 0.001) than low/average OC patients (Table 2). A higher proportion of robotic THAs were performed under spinal anaesthesia compared with conventional THAs (64.3% vs 52.5%, *p* < 0.001).

**Table 2:** Summary of surgical and postoperative factors.

	Overall	Low OC	High OC	*p*
Duration of surgery (minutes), median (IQR)	89 (69–110)	89 (69–108)	89 (67.75–117)	0.261
Anaesthesia type, *n* (%):				
General	816 (45.6%)	529 (39.4%)	287 (64.6%)	**<0.001**
Spinal	972 (54.4%)	815 (60.6%)	157 (35.4%)	
Implant fixation, *n* (%)				
Uncemented	1370 (82%)	1013 (81.4%)	357 (83.8%)	0.054
Cemented	16 (1.0%)	12 (1%)	4 (0.9%)	
Hybrid (cemented femur)	283 (16.9%)	220 (17.7%)	63 (14.8%)	
Reverse hybrid (cemented acetabulum)	2 (0.1%)	0 (0)	2 (0.1%)	
Technique, *n* (%)				
Robotic THA	328 (17.6%)	272 (19.5%)	56 (11.9%)	**<0.001**
Manual THA	1539 (82.4%)	1126 (80.5%)	413 (88.1%)	
Length of stay (hours), median (IQR)	58 (48–98)	56 (36–81)	85 (58–129)	**<0.001**
Length of stay (days), median (IQR)	2 (2–3.5)	2 (1–3)	3 (2–5)	**<0.001**
Postoperative PACU admission, *n* (%)	139 (7.4%)	75 (5.4%)	64 (13.6%)	**<0.001**
30-Day readmission, *n* (%)	113 (6%)	78 (5.6%)	35 (7.5%)	0.146

IQR, inter-quartile range; PACU, Post Anaesthesia Care Unit, percentages may not total 100% due to rounding. Bold indicates statistical significance (*p* < 0.05).

Binary univariate logistic regressions were performed to identify variables for inclusion in the multivariate model, which demonstrated that patients with lower age, manual THA, general anaesthesia, need for PACU admission, higher ASA score, lower preoperative and postoperative Hb, and the year of surgery (later years associated with lower odds of high opioid consumption), were significantly more likely to have high OC when evaluated individually (Table 3).

**Table 3:** Binary univariate regression assessing predictors of higher opioid consumption.

	Odds ratio [95% CI]	*p*
Age	**0.992 [0.985–0.999]**	**0.026**
Male sex	0.822 [0.661–1.022]	0.077
BMI	1.006 [0.991–1.020]	0.440
IMD (quintile)	1.006 [0.925–1.094]	0.887
Manual THA	**1.782 [1.308–2.426]**	**<0.001**
General anaesthesia	**2.816 [2.253–3.521]**	**<0.001**
Need for PACU admission	**2.788 [1.961–3.962]**	**<0.001**
ASA	**1.576 [1.319–1.882]**	**<0.001**
Preoperative Hb	**0.985 [0.978**–**0.991]**	**<0.001**
Postoperative Hb	**0.981 [0.974–0.988]**	**<0.001**
Surgical duration*	0.999 [0.995, 1.003]	0.683
Year of surgery	**0.623 [0.572–0.679]**	**<0.001**

BMI, body mass index; IMD, Index of Multiple Deprivation; PACU, Post Anaesthesia Care Unit; ASA, American Society of Anaesthesiologists Score; Hb, haemoglobin. Bold indicates statistical significance (*p* < 0.05).

* Surgical duration was analysed per 10-minute increment.

**Figure 2:**
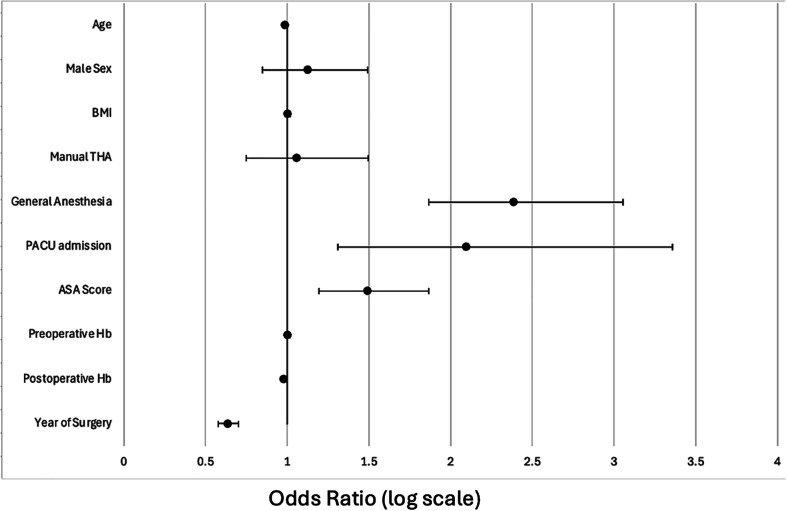
Forest plot showing adjusted odds ratios (95% CI) from the multivariate logistic regression model (*n* = 1,867). Covariates included age, sex, BMI, ASA score, anaesthesia type, PACU admission, haemoglobin levels, surgical technique and year of surgery. The vertical line represents the reference value (OR = 1.0).

After adjusting for baseline demographics in the hierarchical multivariate logistic regression model, several significant independent predictors of higher opioid utilization in the immediate postoperative period were identified (Table 4 and Figure 2). Increased age was found to be associated with a lower likelihood of higher opioid consumption (OR (odds ratio) 0.989 [95% CI 0.981–0.997], *P* = 0.01). Patients who underwent surgery under general anaesthesia (OR 2.386 [95% CI 1.865–3.054], *P* < 0.001) and those requiring PACU admission (OR 2.098 [95% CI 1.310–3.358], *P* = 0.002) were more than twice as likely to have higher opioid consumption. Higher ASA scores were also predictors of having greater opioid consumption (OR 1.492 [95% CI 1.193–1.866], *P* < 0.001). Additionally, lower postoperative Hb (OR 0.981 [95% CI 0.970–0.992], *P* < 0.001) and later year of surgery (OR 0.638 [95% CI 0.579–0.703], *P* < 0.001) were associated with a lower likelihood of higher opioid utilization.

**Table 4:** Multivariate regression assessing predictors of higher opioid consumption.

	Odds ratio [95% CI]	*p*
Age	**0.989 [0.981–0.997]**	**0.010**
Male sex	1.125 [0.847–1.493]	0.418
BMI	1.003 [0.987–1.019]	0.700
Manual THA	1.059 [0.749–1.496]	0.747
General anaesthesia	**2.386 [1.865–3.054]**	**<0.001**
Need for PACU admission	**2.098 [1.310–3.358]**	**0.002**
ASA	**1.492 [1.193–1.866]**	**<0.001**
Preoperative Hb	1.004 [0.992–1.015]	0.314
Postoperative Hb	**0.981 [0.970–0.992]**	**<0.001**
Year of surgery	**0.638 [0.579–0.703]**	**<0.001**

BMI, body mass index; IMD, Index of Multiple Deprivation; PACU, Post Anaesthesia Care Unit; ASA, American Society of Anaesthesiologists Score; Hb, haemoglobin. Bold indicates statistical significance (*p* < 0.05).

## Discussion

The opioid epidemic is a major concern in the field of Orthopaedics, where procedures like total joint arthroplasty contribute significantly to postoperative opioid use. Understanding the factors driving increased opioid consumption is essential to addressing this issue. The purpose of this study was to identify predictors of increased immediate postoperative opioid usage in patients undergoing primary THA. Among 1,867 cases, we identified that higher ASA scores, surgery under general anaesthesia, and the need for postoperative PACU admission were significant independent predictors of increased opioid consumption. Older patients, higher postoperative haemoglobin levels, and a more recent year of surgery were associated with a lower likelihood of high opioid consumption in the immediate postoperative period. The observed temporal reduction in opioid consumption could reflect institutional improvements in enhanced recovery pathways, stricter opioid prescribing stewardship and the increasing use of regional anaesthetic techniques over time. However, as this was an observational study, causality cannot be inferred.

Identifying predictors of increased opioid consumption in THA patients holds significant clinical utility, particularly in developing targeted postoperative protocols and risk stratification tools. Understanding these predictors could allow healthcare providers to anticipate which patients may have higher opioid requirements, enabling proactive, personalized analgesic strategies that can improve pain management outcomes. In an era increasingly focused on personalized medicine [19], such insights are particularly valuable, as they allow tailoring of pain management approaches to individual risk profiles, minimizing opioid use without compromising pain control. This study’s findings may serve as a foundation for refining post-THA care pathways, facilitating efficient use of multimodal analgesia, and reducing reliance on opioids, thereby enhancing both patient satisfaction and functional recovery.

In our cohort, we observed that younger patients consumed more opioids postoperatively compared to older patients. This may be due to healthcare providers prescribing more conservative opioid doses to older patients, who are at higher risk for opioid-related adverse effects and complications [20]. This finding was consistent with other total shoulder [21], hip and knee arthroplasty [20, 22] literature, where younger patients exhibited higher opioid consumption. However, some studies have reported contrasting results, showing that advanced age was associated with prolonged opioid use [23] and that younger patients required smaller quantities of opioids following surgery [24]. In a large database study of over 79,000 patients, Jiang et al.. [25] identified patient ages 40–59 among the highest risk factors for chronic opioid use. These variations suggest that opioid consumption patterns are influenced by a complex interplay of factors, including patient-specific characteristics that may affect individual differences in pain perception or pain management expectations, and provider prescribing practices.

We also identified that higher ASA scores and PACU admission were associated with higher postoperative opioid consumption. This may indicate that patients with more comorbidities and poorer overall health may experience increased pain or have diminished physical resilience in coping with postoperative discomfort, leading to higher opioid requirements. Similarly, patients with higher postoperative haemoglobin levels were less likely to consume higher amounts of opioids, potentially reflecting lower intraoperative blood loss and less surgical trauma, which could contribute to lower pain levels and opioid requirements. These findings align with those of Cao et al., who reported that reducing blood loss with tranexamic acid in total knee arthroplasty was associated with lower opioid consumption [26], suggesting that minimizing surgical blood loss and managing intraoperative factors can mitigate postoperative pain. Early identification of patients with more comorbidities and PACU admissions may help flag those at risk for higher opioid use, enabling timely interventions to optimize analgesia without over-reliance on opioids.

In terms of surgical factors, multivariate analysis found that general anaesthesia was a significant predictor of high opioid consumption in our cohort. This is in keeping with literature demonstrating that regional anaesthetic techniques, offer superior pain relief, reduce MME requirements, and therefore lower the risk of opioid-related adverse drug effects, including nausea, vomiting, and altered mental status [27, 28]. Owen et al. also observed that spinal anaesthesia for THA led to shorter inpatient hospital stays without increasing the risk of postoperative medical complications, readmissions, or reoperations [28]. In a large database study of THA patients, general anaesthesia was found to be associated with longer surgical times and adverse events when compared to spinal anaesthesia [29]. Where feasible, neuraxial anaesthesia should be favoured as part of multimodal perioperative protocols, given its association with lower inpatient opioid requirements and improved recovery profiles. Although the univariate analysis suggested an association between robotic THA and higher opioid use, this did not persist after multivariate adjustment. This may partly reflect differences in case mix and anaesthesia type between groups.

Multimodal analgesic strategies, incorporating non-opioid options such as NSAIDs and regional nerve blocks, have demonstrated effectiveness in reducing opioid consumption for THA patients while maintaining effective pain control [14, 30]. These approaches, now standard in total joint arthroplasty, provide a structured method for achieving pain relief without the risks associated with narcotics. Regional techniques, including neuraxial anaesthesia and peripheral nerve blocks, as part of a multimodal perioperative analgesic plan, have been associated with improved postoperative outcomes and reduced opioid requirements. Incorporating NSAIDs as part of a multimodal regimen has shown efficacy in minimizing postoperative pain and opioid use, and is strongly recommended [22, 31]. A well-rounded multimodal strategy that includes NSAIDs, acetaminophen, gabapentin, local anaesthetics, and/or peripheral nerve blocks can play a vital role in enhancing patient satisfaction and functional recovery following THA.

Adverse effects of opioid use are well known and increase with prolonged postoperative consumption, highlighting the need for strategies that minimize opioid exposure to reduce complications such as misuse, dependence, and even death. The findings of our study highlight opportunities for more personalized pain management strategies in THA patients. For example, younger patients and those requiring general anaesthesia may benefit from enhanced multimodal analgesia protocols that incorporate non-opioid alternatives and regional anaesthesia techniques. Patients with higher ASA scores, higher intraoperative blood loss, or those requiring PACU admission may benefit from proactive monitoring and early intervention to manage pain effectively and reduce opioid reliance. The choice of anaesthesia and the use of tranexamic acid can be carefully considered in high-risk patients to optimize pain management and recovery outcomes. Roebke et al. found that inpatient opioid use within 24 h of discharge was the strongest predictor of 90-day postoperative opioid consumption [22]. General surgery and internal medicine literature have also demonstrated a dose-response relationship between inpatient narcotic use and a higher risk of persistent opioid use, with opioid prescriptions at discharge linked to chronic opioid use [32]. This suggests that prescribers should consider limiting inpatient opioid doses in the immediate postoperative period, and adopt a multimodal pain management approach to reduce long-term opioid risks.

While this study provides valuable insights, several limitations should be considered. As a single-centre analysis, our findings may have limited generalizability to broader populations with varying demographic and clinical profiles. Pain is a complex, multifactorial variable influenced by individual thresholds and subjective experiences. While we accounted for key demographic and surgical factors, pain scores and preoperative opioid exposure data were not available for analysis, both of which may influence postoperative opioid requirements. In addition, psychological factors such as anxiety, depression and pain catastrophising, were not captured in our dataset [33]. However, to account for potentially non-modifiable systemic changes over time – such as non-quantifiable enhancements in perioperative care and opioid-prescribing practices – we included the year of surgery as a variable in the multivariate model. The observed decrease in opioid use over recent years may reflect these systemic improvements in addition to patient factors. Strengths of our study include the large sample size and robust multivariate analysis, allowing for the identification of independent predictors of opioid consumption and helping mitigate the impact of potential confounders.

Our study provides important insights into postoperative opioid use in THA patients, highlighting key predictors such as younger age, higher ASA scores, and general anaesthesia. These findings emphasize the need for tailored, individualized pain management strategies to reduce opioid consumption and minimize the associated risks and adverse effects. By addressing these predictors, healthcare providers can optimize pain relief, improve patient outcomes, and enhance satisfaction. Furthermore, the choice of anaesthesia and surgical technique should be carefully considered to optimize pain outcomes. To further validate and extend these findings, additional corroborative studies are warranted.

## Data Availability

The datasets generated and analyzed in the current study are not publicly available due to data protection regulations. Access to data is limited to the researchers who have obtained permission for data processing. Further inquiries can be made to the corresponding author.
